# Nano-Vesicle (Mis)Communication in Senescence-Related Pathologies

**DOI:** 10.3390/cells9091974

**Published:** 2020-08-26

**Authors:** Sherin Saheera, Ajay Godwin Potnuri, Prasanna Krishnamurthy

**Affiliations:** 1 Department of Cardiovascular Medicine, University of Massachusetts Medical School, Worcester, MA 01605, USA; sheru.rosh@gmail.com; 2 Department of Animal Physiology, Indian Council for Medical Research—National Animal Resource Facility for Biomedical Research, Genome Valley, Shamirpet, Hyderabad, Telangana 500078, India; ajaygodwin@outlook.com; 3 Department of Biomedical Engineering, School of Medicine and School of Engineering, The University of Alabama at Birmingham, 1675 University Blvd, Volker Hall G094, Birmingham, AL 35294, USA

**Keywords:** exosomes, aging, extracellular vesicles, miRNA, COVID-19

## Abstract

Extracellular vesicles are a heterogeneous group of cell-derived membranous structures comprising of exosomes, apoptotic bodies, and microvesicles. Of the extracellular vesicles, exosomes are the most widely sorted and extensively explored for their contents and function. The size of the nanovesicular structures (exosomes) range from 30 to 140 nm and are present in various biological fluids such as saliva, plasma, urine etc. These cargo-laden extracellular vesicles arise from endosome-derived multivesicular bodies and are known to carry proteins and nucleic acids. Exosomes are involved in multiple physiological and pathological processes, including cellular senescence. Exosomes mediate signaling crosstalk and play a critical role in cell–cell communications. Exosomes have evolved as potential biomarkers for aging-related diseases. Aging, a physiological process, involves a progressive decline of function of organs with a loss of homeostasis and increasing probability of illness and death. The review focuses on the classic view of exosome biogenesis, biology, and age-associated changes. Owing to their ability to transport biological information among cells, the review also discusses the interplay of senescent cell-derived exosomes with the aging process, including the susceptibility of the aging population to COVID-19 infections.

## 1. Introduction

Extracellular vesicles (EVs), once considered to be cellular waste products with minimal biological or clinical significance, have evolved over time to be one of the critical mediators of intercellular communications, biomarkers for various diseases, and biovesicles for drug delivery and therapy. Extracellular vesicles are lipid bound and are secreted by different type of cells. They form a heterogeneous group comprising mainly of exosomes, microvesicles, and apoptotic bodies [1]. They are grouped based on their size, biogenesis, composition, and function. The size of microvesicles is approximately 100–1000 nm in diameter and they originate from the outward budding of the plasma membrane. The apoptotic bodies are generated by the blebbing of plasma membrane of cells undergoing apoptosis and the size ranges from 100 to 5000 nm [2,3]. Of the various EVs, exosomes are the smallest (30–140 nm) and the most extensively studied [4]. The membrane bound vesicles are secreted by almost all cell types and have been isolated from mucosal and endogenous biofluids such as blood, urine, tears, lymph, gastric acid, breast milk, and saliva [5]. Although EVs vary in their origin, biogenesis, secretion, targeting, and final fate [6,7], they have been implicated in key processes such as growth and development, cell-to-cell communication, immunomodulation, blood coagulation, aging, and various pathologies [8].

The plasma membrane-derived lipid bilayer of EVs protects within it a diverse cargo of nucleic acids, proteins, and lipids, and they are shielded against degrading enzymes such as nucleases and proteases [9]. These cargos are stable under physicochemical conditions generally considered adverse for biological materials. Moreover, the composition of the EVs represents a snapshot of the cell status at the time of secretion, and studies have reported that pathological states such as cancer, premature senescence, oxidative stress, and apoptosis could alter their composition [10].

## 2. Microvesicles and Apoptotic Bodies

All microvesicles (MVs) have specific ‘marker proteins’ regardless of the cell type from which they are released. Since they are formed by the outward blebbing of the plasma membrane, they mainly consist of cytosolic and plasma membrane-associated proteins such as tetraspanins, cytoskeletal proteins, integrins, and heat shock proteins [6]. MVs target or interact with other cells with the help of glycan-binding proteins on their surface [2]. MVs have the ability to package active cargo (such as nucleic acids, proteins, and RNAs) and deliver it to neighboring cells and can thereby modulate/regulate their function [11,12]. The cargo varies depending on the physiological or pathological state of the cells. Some MVs released by Mesenchymal Stem Cells (MSCs) in response to oxidative stress could also carry mitochondrial particles along with mitochondrial DNA (mtDNA) [13]. Perhaps understanding the composition of MVs could aid in better therapeutic strategies.

Apoptotic bodies are released by dying cells, and they tend to be on the larger side (1–5 µm) based on the size of various EVs [6]. The apoptotic bodies have a very different composition compared to MVs and exosomes. They majorly contain intact organelles, chromatin, histones, and glycosylated proteins.

## 3. Exosomes: Biogenesis and Release

**Figure 1:**
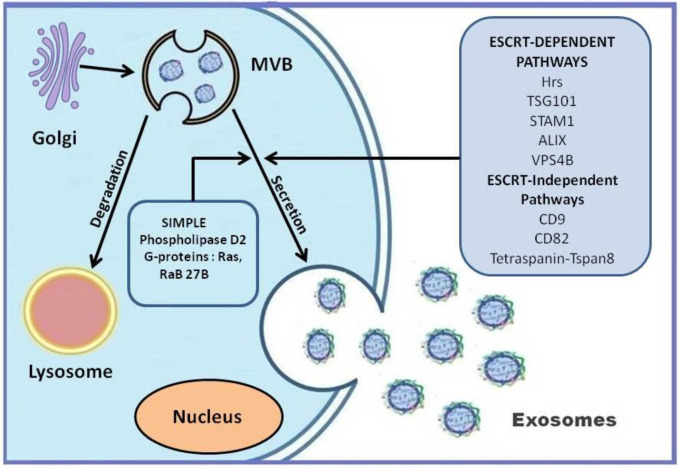
Exosome biogenesis and secretion. The biogenesis of exosomes is mediated by either ESCRT-dependent or ESCRT-independent pathways. ESCRT pathways involve numerous proteins/enzymes. Multivesicular bodies fuse with the ce ll membrane and result in the release of exosomes. (SIMPLE: Small Integral Membrane Protein of the lysosome/late endosome; MVB: Microvesicular Bodies; ESCRT: Endosomal Sorting complex required for Transport; Hrs: Hepatocyte growth factorregulated tyrosine kinase substrate; STAM: Signal Transducing adaptor Molecule; TSG101: Tumor susceptibility gene 101; VSP4B: Vacuolar Protein Sorting 4 Homolog B).

‘Platelet dust’ was the term used for the first time to describe EVs by Wolf [14]. Thereafter, all biological fluids were found to contain vesicles of different sizes [7]. The smallest of all the EVs (less than 150 nm), the exosomes were first visualized in the reticulocytes of rat and sheep [15]. The vesicle release was considered as a mechanism for the elimination of specific membrane proteins like transferrin receptors, which are known to diminish during the maturation of reticulocyte [15]. The exosomes are released during the fusion of microvesicular bodies (MVBs) with the plasma membrane [6]. The release of exosomes has many steps involved such as (i) the formation of intraluminal vesicles in MVBs, (ii) their transport to plasma membrane, and (iii) fusion (Figure 1). Normally, MVBs help in clearing cellular waste by undergoing degradation in the lysosomes. To some extent, the composition of exosomes reflects the composition of MVBs. Hence, exosomes could also carry misfolded and harmful proteins and can contribute to disease progression [16].

Various studies have demonstrated different strategies for the biogenesis of exosomes. One of the broadly accepted strategies is the involvement of the Endosomal Sorting complex required for transport (ESCRT). ESCRT is activated during membrane budding, cytokinesis, and autophagy, and it is involved in EV biogenesis [1]. The exosome biogenesis can occur either via ESCRT-dependent or ESCRT-independent pathways and mainly depends on the type and physiological state of the cell. In addition, the biogenesis pathways will also determine the composition of various exosomes [17]. Figure 1 illustrates the many different proteins involved in the exosome biogenesis.

Exosomes released from different cell types contain different lipids and proteins. The lipid composition of exosomes comprises those lipids that are part of the plasma membrane and the Golgi, and they are enriched in glycosphingolipids, cholesterol [18], phosphatidylserine [19], and ceramide. Exosomal cargo also includes RNAs such as mRNAs, miRNAs, LncRNAs, and circular RNAs as well as short DNA sequences. These cargos are potential mediators of cell survival, homeostasis, cell functions, and intercellular communications. Put together, these distinct characteristics position EVs as a potential class of biomarkers with strong diagnostic potential in the context of personalized medicine.

Exosomes have been implicated in a variety of biological processes. Dendritic cell-derived exosomes have been shown to modulate T-cell response similar to the intact cells, thus implicating an immunomodulatory role for these nanovesicles [20]. Exosomes with hsc73, a heat shock protein, have been reported to elicit antitumor activity [21]. The FasL ligand in exosomes from tumor cells has been shown to mediate apoptosis [22]. The composition and the function of the exosomes vary depending on the cell type and reflects their pathophysiological state. Depending on the protein composition, the exosomes may have varied effects such as cytotoxic, immunomodulatory, and apoptotic activity. Exosomes help transfer membrane proteins between cells without the requirement of direct cell–cell contact [23].

## 4. Senescence and Its Effect on Stem Cell Biology, Transplantation, and Function

Aging is a complex cellular and molecular process involving both genetic and environmental factors. The salient features of aging include elevated Reactive Oxygen Species (ROS) levels, mitochondrial dysfunction, genetic mutations, and DNA damage [24]. With aging, inflammation and macromolecule dysfunction slowly sets in, leading to irreversible damage/senescence of cells [25]. Mitochondrial dysfunction is the major reason for the increased oxidative stress. Adult stem cells have also been shown to exhibit senescent phenotype with increasing age [26,27]. These pathological changes increase the burden on cells, which will ultimately succumb to apoptosis. Studies have shown that a reduction of senescent cells can lead to reduced inflammation, macromolecular dysfunction, and an improvement in progenitor functions [28]. The aging process can be driven by cell autonomous or cell non-autonomous mechanisms. The time-dependent accumulation of damaged macromolecules, genetic material, and organelles can lead to cell autonomous aging [29]. However, studies have shown that circulating factors in younger healthy animals could regenerate the tissues in older mice. Factors such as Growth Differentiation Factor 11 (GDF-11) and oxytocin are some examples and are termed anti-geronic [30]. For instance, recombinant GDF (rGDF) has rejuvenating effects in heart, skeletal muscles, and brain [31]. Conversely, some factors in blood from old patients have been shown to drive aging. Pro-geronic factors such as chemokine CCL-11 and β2 microglobulin could play roles in cell non-autonomous aging [32]. Since EVs are released by all cells, they could act both as anti- and pro-geronic factors.

Senescence involves the loss of the proliferative potential of normally replication-competent cells. Senescent cells usually develop a senescence-associated secretory phenotype (SASP) and are characterized by an increased release of pro-inflammatory cytokines and chemokines, and tissue-damaging proteases [33]. They also released factors that can alter stem and progenitor cell function, hemostatic factors, and growth factors. The markers of senescent cells are an increased expression of cell cycle regulators like p16^INK4A^ and p21Cip1. [34,35]. They also have increased SASP factors such as IL-6, IL-8, monocyte chemoattractant protein-1, and plasminogen-activated inhibitor-1, increased senescence-associated β-galactosidase (SA-βgal) activity and telomere-associated DNA damage foci (TAFs) [36]. These SASP features have substantial pathological effects to the neighboring cells. Studies found that removal of the p16INK4a-positive cells and also clearing senescent cells extend the lifespan in transgenic mice models [37]. On the contrary, injecting senescent cells could drive age-related diseases, indicating the critical role played by these cells in determining the physical condition [38]. Senescent cells have been reported to release more EVs than normal cells, and they exhibit a totally different composition [39]. These EVs could also be considered part of the SASPs and could play role in senescent cell-induced premature aging.

Another effect of aging is the loss of ability of cells to effectively respond to stress and their limited regenerative capacity. Several studies have reported an age-dependent decline in the characteristics of several different types of adult stem cells [40]. Mesenchymal Stem Cells (MSCs) from old mice have been shown to be defective in differentiation [41]. Interestingly, injecting young MSCs into old mice has shown to expand the lifespan [42].

The bone marrow and adipose tissue-derived MSCs were found to have reduced ability to respond to oxidative stress when isolated from aged donors [43]. MSCs are a key component of the hematopoietic niche and with aging, MSCs exert a detrimental effect on the hematopoietic system. Neural stem cells exhibit compromised proliferation and differentiation as well as an enhanced senescence phenotype with aging [44]. Age-associated cognitive effects such as neurodegenerative disorders, memory defects, and olfactory dysfunction were consequent to the deterioration of NSC function. The function of the HSCs is modulated by the microenvironment, which produces the cell-intrinsic and extrinsic factors [45]. However, aging impairs the microenvironment, which alters HSC functions, similar to cell cycle regulation, proliferation, and differentiation. c-kit^+^ Cardiac Stem Cells (CSCs) residing within the myocardium also exhibit age-associated changes. CSCs from aged patients expressed the cyclin-dependent kinase p16^INK4a^ [46]. CSCs from older mice had reduced telomere length and increased apoptosis [47]. The same changes were observed in Wistar rats with aging and researchers also found that the incidence of hypertensive heart disease accelerated the aging process [27].

Several studies indicate the protective effect of EVs from stem/progenitor cells. For instance, EVs from MSCs have been shown to have the ability to repair damaged tissues and can induce healing of liver, kidney, and heart [48,49]. Therefore, one could assume that EVs from senescent cells would have detrimental effects. The EVs act as messengers by delivering their cargo to the target cells and could explain their role as potential pro- and anti-geronic factors.

Cellular aging could also influence the transplantation outcomes. Age-associated changes in the stem cells and how agingimpairs their functional ability are now well understood. Hence, the age of the donor and the recipient are of prime importance during stem cell transplantation. The stem cells from a young recipient could rejuvenate whereas those from an aged donor exert tissue dysfunction [50]. In addition, the recipients’ microenvironment modulates the fate of stem cells being transplanted. When hematopoietic stem cells (HSCs) from old mice were transplanted, they exhibited reduced rates of homing and engraftment along with impaired differentiation ability [51]. When performing hematopoietic cell transplantation, it is critical to assess the chronological as well as biological age of the patient. The biological age of the cells can be attained by evaluating their clonogenic capacity and telomere length [52]. The telomere length of autologous CD34^+^ cells did not have any influence on the clinical outcome in cardiomyopathy patients. However, a higher CD34^+^ human Telomerase reverse Transcriptase (hTERT) expression was associated with better clinical outcome [53]. A study found that the increasing age is associated with low peripheral blood CD34^+^ cells and diminished bone marrow stem cell mobilization by Granulocyte Colony Stimulating Factor (GCSF) in ischemic heart failure patients [54]. EVs secreted from both the transplanted and recipient cells could modulate the cellular behavior and modify signaling pathways. EVs from young stem cells were found to have higher amounts of galectin-3 and certain miRNAs that promote stem cell function compared to those from senescent cells [55]. EVs from senescent cells accelerated age-associated processes such as inflammatory gene expression and telomere dysfunction.

## 5. Role of Extracellular Vesicles in Age-Related Diseases

**Figure 2:**
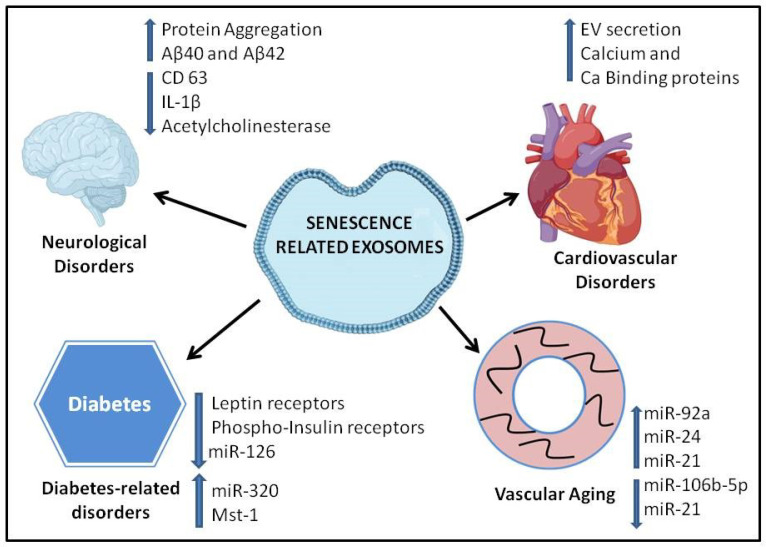
Age-related complications of senescent cell derived exosomes. Extracellular vesicles (EVs) secreted from senescent cells have been implicated in cardiovascular diseases, diabetes, neurological disorders, and vascular aging. Senescent EVs modulate several different proteins and miRs, thus exacerbating age-associated pathologies.

Aging is an independent risk factor in the development of neurodegenerative disorders, cardiovascular disease, and diabetes, which are the primary causes of mortality and morbidity in the elderly population [56]. Most recently, with regard to the COVID-19 (Coronavirus disease 2019) pandemic, the aging/elderly population is known to be extremely vulnerable to the disease, resulting in a higher fatality rate [57,58,59]. Age-related changes occur at the cellular and molecular level, affecting the physiological function of tissues and organs. The senescent cells are characterized by cell growth arrest and altered differentiation functions [60]. All these changes result from the cumulative effect of many signaling pathways regulating different cellular processes in the cell. The accumulating evidence suggests that exosomes are physiologically relevant intercellular messengers [6]. Senescence-associated exosomes could transfer many molecules and could accelerate the aging process or associated pathologies in an autocrine, paracrine, and endocrine fashion [61] (Figure 2). A study was carried out to understand the premature cellular senescence and alterations in exosome biogenesis during irradiation in human prostate cancer cells. This analysis identified a novel connection between the induction of p53-dependent senescence and the release of exosomes [61]. Importantly, this supports the possibility that senescence-associated exosomes can transfer cargos between cells that may be recruited to increase the exosome release observed during cellular senescence. EVs from older individuals were shown to have MHC-II expression on monocytes, which was indicative of the effect of EVs on modulating immune response [62]. These EVs are taken up faster by B cells in older individuals when compared to young, and as a result, the levels of circulating EVs could be reduced. This is in contrast to the observation of increased EV production in aged cultured cells [29].

## 6. Neurodegenerative Disorders

In neurodegenerative diseases such as age-related macular degeneration (AMD) and Alzheimer’s disease (AD), exosomes have been implicated [63]. The contribution of extracellular vesicles (EVs) to peripheral inflammation during aging is also reported [64]. A previous study involving young and old Wistar rats suggests that the normal aging process adversely changes the profile of central and circulating extracellular vesicles. The study aimed at determining the protein concentration, CD63 content, along with AChE activity, in plasma and Cerebrospinal Fluid (CSF) from 3- and 21-month-old Wistar rats [65]. This study correlates the systemic inflammation widely described in the aging process and the susceptibility to age-related diseases such as atherosclerosis and diabetes. During the process of physiological aging, chronic inflammation characterized by increased pro-inflammatory cytokines ensues. The study evaluates exosomal inflammatory cytokines in the healthy aging process. The study concluded that healthy aging changes circulating EV profile in rodents with significant change in the expression of exosomal marker such as CD63 and Acetylcholinesterase (AChE) activity [65]. Platelet derived EVs also carry cytokines, such as IL-1β and inflammasome components in the synovial fluid, as evidenced in patients with rheumatoid arthritis [66]. Interestingly, EVs from the plasma of aged rats show reduced IL-1β levels, which is consistent with the exosome profile in inflammatory condition such as rheumatoid arthritis [25]. The altered IL-1β levels in circulating EVs can be linked to age-related inflammatory conditions. In addition, the disruption of the CSF exosomes as assessed by lower CD63 levels can be related to susceptibility to neurodegenerative disorders in the elderly. It has been observed that the EVs in most inflammatory diseases carry within them damage-associated mediators, cytokines, autoantigens, and tissue-degrading enzymes [67]. Endothelial toxins such as Aβ40 and Aβ42 were observed in the plasma endothelial-derived exosomes of patients with Alzheimer’s disease [68]. Activated microglia could mediate neuroinflammation in various neuropathologies such as Parkinson’s disease [69]. Misfolded alpha-synuclein (α-syn) can further induce misfolding of the α-syn, leading to protein aggregation in neurons, thereby mediating inflammation. Studies have found that α-syn can spread between neurons through exosomes [70]. Astrocytes-derived exosomes have also been shown to induce protein aggregation in the brain [71].

## 7. Cardiovascular Disorders

A general feature of cellular senescence is increased EVs secretion [29]. Various stimuli such as serial passaging, senescence, and cell damaging processes such as irradiation and DNA-damaging reagents enhance EVs secretion [36]. A recent study on microvesicles from the plasma of senescent Endothelial Cells (ECs) promoted the calcification of human aortic smooth muscle cells [11]. A previous study has reported the presence of senescent ECs in human atherosclerotic plaque [11], suggesting a specific role of senescent MVs in plaque development. Along with oxidative stress, endothelial dysfunction, and inflammation, senescence-associated MVs are considered to be a significant contributor to the development of atherosclerosis [39]. The number of MVs in plasma increases with aging, and they also contain increased amounts of calcium and calcium-binding proteins involved in the calcification of the vessels [72]. Hence, MVs could be used as biomarkers of calcium mineral deposits. MVs might serve as a therapeutic target for the age-associated CVDs such as atherosclerosis, and their quantification and evaluation will help identify patients at risk of CVDs. EVs from senescent cells can mediate early senescence in the neighboring target cell. For instance, EVs from patients with acute coronary syndrome developed early endothelial dysfunction, oxidative stress, premature senescence, and thrombogenicity [73]. miRNAs involved in pathophysiological conditions of the heart are mostly packaged and transported via exosomes [74]. For instance, miR-21 levels were significantly high in patients with aortic stenosis [75]. During heart failure, miR-1, miR-133, miR-208, and miR-499 were found to be enriched in cardiac muscle [76]. Other miRNAs such as miR-1 and miR-133a are also elevated in other cardiovascular diseases [77]. In vitro experiments have shown that cardiac fibroblasts release miRNA-enriched exosomes and facilitate the expression of genes involved in hypertrophy [78]. Next-generation RNA sequencing found that the miRNAs of the exosomes in hypertensive rats were differentially altered compared to normal rats, mainly in relation to the hypertension-specific signaling pathways [79]. The studies indicate that the selective packaging of miRNAs in exosomes under pathological condition could facilitate the development of better diagnosis and treatment for cardiovascular diseases.

## 8. Diabetes

Diabetes mellitus (DM) is a commonly occurring metabolic disorder whose incidence increases with aging. Initial studies have found elevated levels of different cell-derived large extracellular vesicles in individuals with diabetes mellitus. EVs derived from endothelial cells, monocytes, and platelets could be used as a biomarker for DM [80]. However, cell-specific differences in EV production have been reported in diabetes. For instance, erythrocyte-derived EVs were higher, whereas EVs from platelets and leukocytes were not significantly different in diabetic individuals [81]. Several factors such as insulin resistance, body composition, inflammation, diets, drug, and exercise could induce a higher production of EVs in hyperglycemic condition. The diabetic condition also affects the composition of exosomes [82]. EVs from diabetic patients have been found to express lower levels of leptin receptors and phospho-insulin receptors [81].

A study by Wu et al. on EVs from diabetic patients found that they have altered cargo and modulated the morphology and migration of endothelial cells compared to EVs from euglycemic individuals. Chemoattractants such as Vascular Endothelial Growth Factor A (VEGF-A) present in this cargo induce proangiogenic behavior in endothelial cells. These EVs also have inflammatory proteins such as CD40 and HGF, which could have a protective effect in diabetic individuals [83]. EVs play a significant role in contributing to the development of diabetic cardiomyopathy. EVs derived from the cardiomyocytes of diabetic rats expressed higher levels of miR-320 and low levels of miR-126 [84]. These miRs modulate endothelial cell functions such as proliferation, migration, and tube formation. Hsp70-containing EVs from diabetic rats inhibited cardiomyocyte proliferation which otherwise exhibited protection [85]. Another protein from diabetic endothelial EVs, Mst1, promoted the apoptosis of cardiomyocytes [86]. Rats fed with a high-fat diet exhibited increased circulating microvesicles, which had pro-inflammatory effects on endothelial cells [87]. These studies indicate that the intercellular transfer of harmful molecules of EVs between cardiac cells could deteriorate diabetic cardiomyopathy.

Adipokines are biologically active molecules secreted by adipose tissue, and their alterations could lead to metabolic disorders. The dysregulation of adipocyte secretome is linked to the pathophysiology of type-2 diabetes, and this secretome was mostly associated with the exosomes [88]. The exosomes derived from adipocytes primarily act as regulators of inflammation and systemic insulin resistance. In obesity, the altered adipokine composition contributes to the development of metabolic disorders [89]. Adipocytes release fatty acids and other lipids to meet the systemic metabolic needs, and these bioactive molecules are packaged in exosomes. These lipids are transported to local macrophages. The lipid content in the exosomes from obese mice was much higher than that from the lean mice [90]. In pregnant women with preeclampsia or gestational diabetes mellitus, the concentration of placenta-derived exosomes was found to be higher [91]. These observations indicate the role that exosomes play in regulating metabolic disorders.

## 9. Other Age-Related Pathologies

Vascular aging involves phenotypic and structural changes in the vascular wall, which consist mainly of endothelial cells and vascular smooth muscle cells. Studies have found the involvement of various exosomal miRNAs and long non-coding RNAs in EC proliferation, inflammation, angiogenesis, senescence, and apoptosis [92]. For instance, cardiomyocyte-derived exosomal miR-17, miR-19, and miR-126 promoted the proliferation and migration of ECs, whereas miR-92a, miR-24, and miR-21 inhibited the processes [93]. miR-92a and miR-21 are also implicated in EC inflammation by activating inflammatory cytokines and chemokines [94]. Exosomal miRNAs can either act as pro-angiogenic or anti-angiogenic factors. Exosomal miR-125a and miR-106b-5p promote and inhibit angiogenesis, respectively [95]. Exosomes play a critical role in transferring signaling molecules between ECs and VSMCs and thereby modulate vascular aging [92].

With aging, the exosomes from bone marrow interstitial fluid were found to have an altered miRNA profile compared to those from young mice. The miRNA-183 cluster is highly expressed in exosomes from aged mice [96]. This altered expression could affect the osteogenic differentiation and also induce stem cell senescence.

## 10. Exosome Cargo in Senescence

The cells assume a novel phenotype called the senescence-associated secretory phenotype (SASP) when they become senescent, which is characterized by the secretion of a myriad of factors, including the release of exosomes [33]. The secreted factors mediate senescence in cells in the immediate vicinity, which could be detrimental to normal neighboring cells. They do so by blocking growth factor signaling and thereby contributing to the induction of senescence [36]. High levels of exosomes from senescent cells are able to modulate the cellular microenvironment. Exosomes are secreted by most cell types and interact with surrounding cells by introducing regulatory secreted factors or receptors, providing intercellular communication [97]. Exosomes participate in the traffic of protein, lipids, and RNAs to neighboring cells, which are necessary for the rapid phenotype variations.

**Figure 3:**
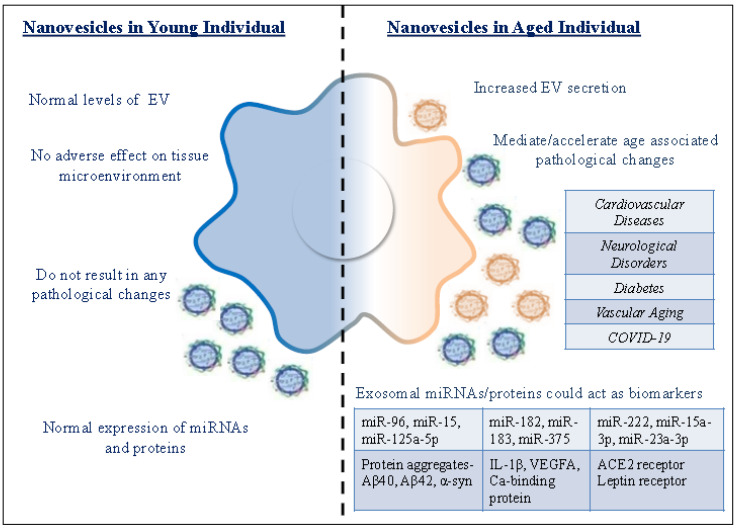
Graphical representation of the age-associated characteristics of nanovesicles.

Senescence is associated with the increased release of exosomes, as observed in normal human fibroblasts [98]. Senescence-associated exosomes were also observed to be released from human-senescent prostate cancer cells [61]. Exosomes released by EGFR-bearing tumor cells are taken up by neighboring endothelial cells and can accelerate the growth of the tumor cell [61]. These studies suggest that senescence-associated exosomes can transfer cargos with both immunoregulatory potential and genetic information and could influence the microenvironment. Several studies have conferred a supporting role for EVs in various diseases and could assist as diagnostic tools. The modulation of senescent cells, senescence-associated factors, and senescent EVs seems to be a promising strategy for mitigating age-related diseases [99]. Salient features of exosomes from young and aged individuals are explained in Table 1 and graphically represented in Figure 3.

## 11. EVs as Potential Diagnostic Markers and Therapeutic Tools for Age-Related Diseases

The EVs can act as biomarkers for various diseases, and they could also indicate the physiological state of the cell/tissue from which they were released. Exosomes are easily accessible in body fluids such as blood, plasma, and urine, making them attractive for use as biomarkers. EVs can also be a representation of the aged phenotype of the cells. EVs has been described as potential non-invasive biomarkers in cardiovascular and inflammatory diseases [100].

EVs from the bone marrow of aged mice have increased miR-96, miR-182, and miR-183, which are part of cluster miR-183. An miRNA-183-5p mimic was shown to increase senescence in bone marrow stem cells [101]. The miRNAs delivered by the EVs are found to be critical regulators in many pathological conditions compared to other cargo molecules such as proteins and lipids [12]. A pilot study on the possible role of salivary exosomal miRNAs as aging biomarkers revealed miR-24-3p to be a novel candidate. The target genes of miR-24-3p activate Mitogen Activated Protein Kinase (MAPK) signaling pathways involved in inflammatory cytokine and chemokine gene regulation [102]. With aging, the increased levels of miR-24-3p may contribute to increased susceptibility to age-dependent alterations in the immune and inflammatory status [103]. Previous studies have examined the relationship between miRNA profiles and aging. A clinical study reported that serum expression levels of miR-151a-3p, miR-181a-5p and miR-1248 were significantly lower in aged humans compared to those in young [104]. Yet another clinical study using serum samples identified five down-regulated miRNAs (miR-29b, miR-106b, miR-130b, miR-142-5p, and miR-340) and three up-regulated miRNAs (miR-92a, miR-222, and miR-375) with aging [105]. These studies support the notion that circulating miRNAs are useful as aging biomarkers. The sensitivity of miRNA amplification from biological fluids can be improved by exosomes isolation, thus making exosomal miRNAs a potential biomarker.

Urinary EVs could be used as biomarkers to identify diabetic nephropathy. Urinary EVs express high levels of C-megalin, which is an endocytic receptor, and they could serve as a potential biomarker [106]. The miRNA profiles of urinary EVs has identified the miR192 and miR-15 family to be differentially expressed, and they could serve as biomarkers.

Diabetic patients have been found to have increased levels of miR-15a-3p in the circulating exosomes isolated from their blood. The up-regulated miRNA was found to inhibit diabetic wound repair by activating the NADPH oxidase 5 (NOX5). The inhibition of circulating exosomal miR-15a-3p was found to accelerate diabetic wound repair and provides a novel target for treating diabetic foot ulcers [107].

EVs in patients with neurological disorders were found to have altered genetic cargo in the form of miRNAs and tau proteins, thus acting as disease biomarkers [108]. EVs were found to have increased levels of p181-tau and p231-tau in the blood of patients with preclinical Alzheimer’s disease [109]. Studies have found that miR-125a-5p, miR-23a-3p, and miR-375 were found to be differentially expressed and could act as biomarkers for Alzheimer’s disease [110]. Differentially expressed exosomal miRNAs as biomarkers for aging are detailed in Table 2.

Exosomes, owing to their low immunogenicity and other beneficial effects, could be utilized for therapeutics or as delivery vehicles. EVs from adipose-derived stem cells have been reported to improve metabolic homeostasis by inducing the production of anti-inflammatory cytokines [111]. Thus, EVs from stem cells might have the potential to improve glucose tolerance in diabetic individuals [111].

## 12. Role of Exosomes in COVID-19 Patients

COVID-19 diseases have by far affected the elderly population more than any other age group, and people over the age of 65 were found to be more susceptible to the infection (WHO COVID-19 dashboard). People with underlying conditions are prone to succumb to the complications compared to healthy individuals [113]. COVID-19 infections result in a multitude of damages in almost all organs—mainly in the lungs and heart.

Recently, researchers found that Angiotensin Converting Enzyme 2 (ACE2) serves as the receptor for the entry of coronavirus into the cells [114]. Studies also were conducted in elucidating the role played by exosomes in the spread of the virus. Exosomes were reported to transfer the ACE2 receptor to recipient cells, and this could also serve as a pathway for the virus internalization and infection. A study found that the components of viruses could be internalized and transferred via caveolin-1 dependent endocytosis or via other extracellular vesicles [115]. Exosomes from virus-infected cells were found to elicit immune response in non-infected cells. Understanding the molecular interactions of miRNA during host–virus interactions will help in the development of effective antiviral therapy. Studies have investigated the various host–cellular miRNAs that play critical roles in viral biogenesis, entrance, replication, and infection [112]. Increasing the levels of host miRNAs could block the entry and propagation of the virus. hsa-miR-203-3p and hsa-miR-4482-3p have been found to target the Spike (S) protein that plays a role in viral replication, whereas hsa-miR-3672 targets the Envelope (E) protein that affects viral envelope formation [112].

## 13. Future Perspectives and Conclusions

The varied nature of the molecules packaged and delivered by exosomes makes it a valuable biomarker for identifying and tracking disease progression and aging. Understanding the relation of aging process to age-related diseases is of great clinical importance for the development of novel therapeutic strategies. The crosstalk of exosomes from the senescent cells with the neighboring cells and its microenvironment has not been well understood. From the studies, it is clear that senescent cell-derived exosomes might be a potential target for age-related therapies and can be achieved by modulating their cargo, mainly miRNAs. Compared to other conventional treatments, the fact that exosomes are small, potent, and non-living makes them highly attractive bioactive molecules. Furthermore, exosomes have the additional advantage that these nanovesicles do not invoke an immune response and also could be used to develop personalized medicines.

## Figures and Tables

**Table 1:** Salient features of exosomes from young vs. aging individuals.

Exosomes from Young Individual	Exosomes from Aged Individual
Normal levels of EV	Increased EV secretion
Exosomes do not have an adverse effect on tissue microenvironment	Exosomes from senescent cells are detrimental to the tissue microenvironment
Exosomal cargo do not result in any pathological changes	Exosomal cargo can mediate/accelerate pathological changes
Normal expression of miRNAs and proteins	Differential expression of miRNAs and proteins could act as biomarkers for diagnosis of age-related pathologies

**Table 2:** Differentially expressed exosomal miRNAs as biomarkers for aging.

Exosomal miRNA	Derived From	Function	Reference
miR-96, miR-182, miR-183	Bone marrow	Increase senescence in bone marrow cells	[101]
miR-24-3p	Saliva	Inflammatory cytokine and chemokine gene regulation	[102]
miR-151a-3p, miR-181a-5p, miR-1258, miR-29b, miR-106b, miR-130b. miR-142-5p, miR-340	Serum	Down-regulated with aging	[104]
miR-92a, miR-222, miR-192	Serum	Up-regulated with aging	[105]
miR-15	Urine	Differentially expressed in diabetic nephropathy	[106]
miR-15a-3p	Blood	Up-regulated in diabetic patients	[107]
miR-125a-5p, miR-23a-3p, miR-375	Blood	Differentially expressed in Alzhemier’s Disease	[110]
hsa-miR-203-3p, hsa-miR-4482-3p,hsa-miR-44366b-3p	COVID-19 patients	Target S protein involved in viral replication	[112]
hsa-miR-190a-5p	COVID-19 patients	Target ORF6 involved in immune suppression	[112]
